# Chemical and Enzymatic Synthesis of Biobased Xylo-Oligosaccharides and Fermentable Sugars from Wheat Straw for Food Applications

**DOI:** 10.3390/polym14071336

**Published:** 2022-03-25

**Authors:** Gabriela Precup, Joachim Venus, Monika Heiermann, Roland Schneider, Ioana Delia Pop, Dan Cristian Vodnar

**Affiliations:** 1 Faculty of Food Science and Technology, Institute of Life Sciences, University of Agricultural Sciences and Veterinary Medicine, 400372 Cluj-Napoca, Romania; gabriela.precup@usamvcluj.ro; 2 Leibniz Institute for Agricultural Engineering & Bioeconomy, Max-Eyth-Allee 100, 14469 Potsdam, Germany; jvenus@atb-potsdam.de (J.V.); mheiermann@atb-potsdam.de (M.H.); rschneider@atb-potsdam.de (R.S.); 3 Department of Exact Sciences, Horticulture Faculty, University of Agricultural Sciences and Veterinary Medicine, Cluj-Napoca, Calea Mănăştur 3-5, 400372 Cluj-Napoca, Romania; popioana@usamvcluj.ro

**Keywords:** wheat straw, monosaccharides, xylo-oligosaccharides, hydrothermal pretreatment, enzymatic hydrolysis, downstream process, food application

## Abstract

Xylo-oligosaccharides are sugar oligomers with 2~7 xylose units considered non-digestible fibers that can be produced from biodegradable and low-cost biomass like wheat straw. An integrated approach consisting of hydrothermal pretreatment, alkaline treatment, enzymatic treatment and the combinations thereof was applied to overcome the recalcitrance structure of the wheat straw and allow selective fractioning into fermentable sugars and xylo-oligosaccharides. The hydrolysates and processed solids were chemically characterized by High-performance liquid chromatography and Ion chromatography, and the results were expressed as function of the severity factor and statistically interpreted. The concentration of fermentable sugars (glucose, xylose, arabinose) was the highest after the combination of alkaline and enzymatic treatment with xylanase (18 g/L sugars), while xylo-oligosaccharides (xylotriose and xylotetraose) were released in lower amounts (1.33 g/L) after the same treatment. Refining experiments were carried out to obtain a purified fraction by using anion and cation exchange chromatography. The polymer adsorber resin MN-502 showed efficient removal of salts, phenols and furan derivatives. However, the xylo-oligosaccharides yields were also slightly reduced. Although still requiring further optimization of the treatments to obtain higher purified oligomer yields, the results provide information on the production of xylo-oligosaccharides and fermentable sugars from wheat straw for potential use in food applications.

## 1. Introduction

Oligosaccharides have attained a growing commercial interest in the last decades due to their application as food ingredients and putative prebiotic compounds, which might exert beneficial effects on the host health [1,2,3,4,5,6,7,8,9]. Some of these polymers, specifically xylo-oligosaccharides (XOS), are considered non-digestible fibers and can be produced from biodegradable and low-cost agricultural biomass, especially lignocellulosic material such as wheat straw, by chemical, auto-hydrolytic, enzymatic processes or a combination thereof [2,4,10,11,12,13,14,15,16,17,18,19,20,21,22].

Xylo-oligosaccharide products are sugar oligomers with 2~7 xylose units, and their monomer (xylose) is connected by β-(1–4)-linkages, which can contain different side groups (e.g., α-d-glucopyranosyl uronic acid or its 4-*O*-methyl derivative, acetyl groups, or arabinofuranosyl residues), forming branched structures [23]. Associations in the scientific literature were made between consumption of XOS and potential prebiotic effects since these compounds pass through the upper gastrointestinal tract without being digested. They are metabolized by lactic acid bacteria (bifido bacteria and lactobacilli) in the lower intestine, which facilitates their conversion into short-chain fatty acids (SCFAs) like acetate, propionate and butyrate. Lately, research has highlighted the implications of the gut microbiota on human health, with SCFAs being correlated with reducing luminal pH and having protective action against acid-sensitive enteropathogens [24,25,26,27,28]. In recent years, XOS have been incorporated in various food applications (cookies, dairy products, beverages, fruit juices and chewing gums) [29,30,31,32], animal feed [33] and nutraceuticals [16,34], showing better physicochemical properties such as thermal stability (up to 100 °C) and acidity (pH 2.5 to 8) compared to the well-known inulin and fructooligosaccharides [5,12,35]. In Japan, XOS are approved as food ingredients by the Japanese Ministry of Health, Labour and Welfare, as Foods for Specified Health Uses (FOSHU), while in China they have been on the market since 2000 as food supplements [36]. In the European Union (EU), XOS obtained from corncobs have been authorized to be used as a novel food ingredient in various food categories (bread, breakfast cereals, biscuits, yoghurt, soy drink, fruit spreads and chocolate confectionary) at maximum levels varying from 3.5 g/kg to 30 g/kg, by the European Commission, as proved by the Commission Implementing Regulation (EU) 2018/1648 of 29 October 2018, based on the scientific opinion on the safety of XOS by the European Food Safety Authority [37,38]. In the EU, foodstuffs produced with new technologies, derived from new sources, and made from new substances, as well as traditional foods consumed in non-EU countries that were not consumed to a significant degree within the EU before 15 May 1997, are considered “novel foods” in Regulation (EU) 2015/2283; therefore, they require premarket authorization by the European Comission after their safety is assessed by the European Food Safety Authority [39,40].

Over the last years, various processes (enzymatic, chemical and autohydrolysis) have been exploited to produce XOS and fermentable sugars from lignocellulosic biomasses such as wheat by-products (straw, chaff and bran) [2,3,10,13,14,15,17,18,19,20,41,42,43], corn cobs [44], almond shells [20], olive stones [20], rice husks [44], barley straw [44], sugarcane straw [45] and poplar wood chips [46]. The state of the art in the manufacture and application of XOS up to 2019 was reviewed recently [10]. The use of agricultural biomass has a positive impact on the environment, by contributing to the valorization of the residues generated annually; however, there are some challenges arising for XOS production, since they present a spoilage risk during storage, are seasonal and have variable chemical composition [47,48]. Wheat straw (WS) is the third most produced cereal in the world, after maize and rice, being a promising feedstock for high value-added products [42]. It was shown to have good xylan/lignin ratios, be biodegradable and have low-cost, but owing an inherent complex structure of the biopolymers cellulose, hemicellulose and lignin in different proportions, a strong native recalcitrance is formed, which blocks its enzymatic hydrolysis [10,21]. Therefore, an integrated approach that would allow selective fractionation of the wheat straw seems a crucial step. Generally, XOS can be produced from xylan, which is the main hemicellulose in wheat straw, consisting of a linear backbone of β-1,4-linked xylopyranose (Xyl) residues. A first pretreatment step of the wheat straw, to break down the complex polymeric structure, and enhancing the accessibility of enzymes to the substrate during a enzymatic hydrolysis step were shown to be effective for generating fermentable sugars and XOS [6,10]. Previous studies showed that autohydrolysis, or hydrothermal pretreatment (HTP), might be an attractive method, since it was low-cost and used only water as a reaction media, effectively depolymerizing hemicelluloses by the hydrolytic action of hydronium ions (generated from water autoionization and from in situ generated organic acids) into soluble sugars and XOS and enhancing the accessibility of enzymes to the solid fraction (cellulose and lignin) [10,15,34]. The extent of depolymerization depends on the treatment severity (temperature and time), with studies showing a varied range of temperatures as effective (130–230 °C), but also on other factors such as particle size, pH and liquid-solid ratio [18]. To remove the lignin from the pretreated residues, post-treatment technologies are used, such as alkaline, acidic and enzymatic processes or a combination thereof. Alkali treatment was shown to improve enzymatic digestibility, by degrading the lignin structure and swelling the cellulose fibers [10,49]. However, the chemical and autohydrolysis methods showed shortcomings, such as degradation of pentoses to contaminants like furfural and hexoses to hydroxymethylfurfural (HMF), low control over the degree of polymerization (DP) and high downstream costs [50]. A second step consisting of applying an enzymatic treatment for xylan hydrolysis, using endo-1,4-β-xylanases (EC 3.2.1.8) and endo-1,3-β-xylanases (EC3.2.1.32), affected the yield of XOS, depending on various factors such as enzyme activity and incubation conditions (pH, hydrolysis time and temperature) [4,12]. The enzymatic process was reported to be more environmentally friendly, since no use of chemicals is needed, and it can be conducted at milder temperatures; however, the cost of the various enzymes needed might be quite high.

**Figure 1:**
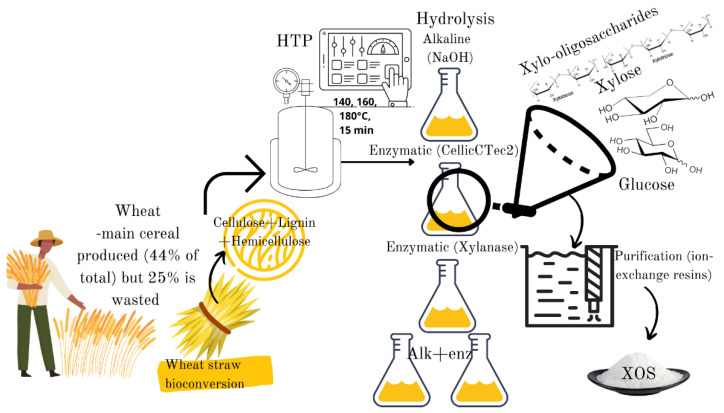
Graphical abstract: Chemical and enzymatic synthesis of biobased xylo-oligosaccharides and fermentable sugars from wheat straw.

Against this background, the main objectives of this paper were to characterize the liquid fractions of wheat straw obtained during the autohydrolysis, alkali and enzymatic treatment and the combinations thereof in terms of XOS production (xylobiose, xylotriose and xylotetraose) and fermentable sugars. A downstream process method consisting of filtration, decolorization, and an- and cation exchange resins was tested in order to obtain a purified fraction of oligosaccharides and fermentable sugars for potential use in food applications (Figure 1).

## 2. Materials and Methods

### 2.1. Material

Wheat straw was kindly provided from a farm (SC ALBATROS SRL) in Romania. The feedstock material was ground with a knife mill to particles of 0.5 mm, homogenized in a defined lot and stored in plastic containers at room temperature.

### 2.2. Hydrothermal Pretreatment (HTP)

The hydrothermal pretreatments (autohydrolysis) were performed according to Budde (2014), in a stainless-steel reactor (Parr Instruments Company, Moline, IL, USA) with a total volume of 600 mL [51]. The reactor was fitted with two four-blade turbine impellers, heated by an external fabric mantle and cooled down with ice water manually. Temperature was controlled through a Parr PID controller, model 4843.

The wheat straw was mixed with water in the reactor in order to obtain a L/S ratio of 10 (g water/g dry feedstock). The agitation speed was set at 350 rpm, and the reactor was heated to reach final temperatures ranging between 140 °C and 180 °C for 15 min (isothermal conditions). Three repetitions were made for each batch. Typically, the average heating rate (from 100 °C) was 3.8 °C/min. When the desired temperature was attained, the reactor was maintained for a reaction time of 15 min, and then it was rapidly cooled down to 70 °C. The liquid and solid phases were recovered by filtration (Whatman filter paper No. 1), and the whole slurry was filtered for solid and liquid recovery. After the filtration, the liquid phase was collected in a plastic vial and stored in a refrigerator at 4 °C for pH measurement as well as sugar and byproduct analyses. The solid residues were washed under tap water and centrifuged and stored in a refrigerator at 4 °C for enzymatic hydrolysis.

The severity of autohydrolysis pretreatment is often expressed in the literature as the “severity factor”, which refers to the combination of temperature and residence time in the autohydrolysis pretreatment process. It was calculated using the following equation [52]:

(1) $$Severity\;factor=\log_{10}[t_{1}\;\times \;\exp\;(T_{1}-100/14.75)]$$

where t_1_ and T_1_ are the pretreatment time (min) and temperature (°C), respectively. The value of 14.75 is an empirical parameter related to temperature and activation energy. Severity factor was calculated in the range of 2.35–3.53, depending on pretreatment temperature and time.

### 2.3. Chemicals

Glucose (G), xylose (X), arabinose (A) and standards were acquired from Sigma-Aldrich Co. (St. Louis, MO, USA) to determine the chemical composition of wheat straw and monomeric sugars in the liquid fraction. The xylo-oligosaccharides standards (X2) xylobiose, (X3) xylotriose and (X4) xylotetraose were acquired from Megazyme International (Ireland). All of the reagents were of analytical grade.

### 2.4. Analytical Methods

#### 2.4.1. Chemical Characterization of Feedstock and Processed Solids

The materials were ground in a knife mill to a particle size of 0.5 mm, and the moisture content was determined by oven-drying at 105 °C to a constant weight. The ash content and organic dry matter was determined by igniting the contents at 550 °C for 5 h in a muffle furnace. The main components of the wheat straw were determined using the laboratory analytical protocol (LAP) developed by the National Renewable Energy Laboratory (NREL).

#### 2.4.2. Chemical Characterization of Hydrolysates

Glucose, xylose, arabinose, acetic acid, 5-hydroxymethylfurfural (HMF) and furfural were analyzed by HPLC (Waters, Milford, DE, USA) using an Aminex HPX-87P column (Bio-Rad, Hercules, CA, USA) and a Shodex sugar SP0810 column. All samples were filtered through 0.2 μm membranes before analysis. A sample of the liquors was directly analyzed by HPLC. The yields of cellulose in the liquid phase were calculated based on Equation (2), with 0.9 being the conversion factors for glucose.

(2) $$Cellulose\;yield\;\left(\%\right)=\frac{Total\;glucose\;released\;\left(g\right)\times 0.9}{sample\;dry\;weight\;\left(g\right)}\;100.\;$$

#### 2.4.3. Enzyme Assays

##### Cellulase Assay

The cellulase activity was assayed by measuring the amount of reducing sugars released from the wheat straw according to Ghose (1987) and recommended by IUPAC, using filter paper Whatman No. 1 as a substrate [53]. A sodium citrate buffer solution of 0.05 M and pH 4.8 was prepared, of which 1 mL was added in a glass tube, covering the paper strip together with 0.5 mL of enzyme. The tube was vortexed and incubated in a water bath at 50 °C for 60 min. Then, 3 mL of DNS reagent was added at the end of the incubation, and the tubes were incubated in a water bath for 10 min at 95 °C and then placed in a cold-water bath for 5 min. Dilutions were made, and the absorbance was measured at 540 nm against a reagent blank using an LLG–uniSPEC 2 spectrophotometer. The amount of reducing sugar liberated was determined by the Dinitrosalicylic (DNS) acid method, using glucose as the standard. One FPU/g represents the enzyme unit per gram of the initial dry solid substrate.

##### Xylanase Assay

The crude endo-β-1-4-xylanase used in this study was produced from *Trichoderma viride* and provided by Sigma-Aldrich (USA). The reaction mixture containing equal volumes of 1% (*w*/*v*) beechwood xylan and the suitably diluted enzyme solution with 50 mM phosphate buffer (pH 7) at 37 °C for 30 min, allowing depolymerization of xylan. The reaction was stopped by adding 1500 μL DNS reagent, and then the mixture was heated at 100 °C for 10 min, allowing the color-forming reaction. The intensity of red-brown color was measured at 515 nm to estimate the concentration of reducing sugar in the reaction system. One xylanase unit was defined as the amount of enzyme needed for the liberation of one µM of reducing sugar (xylose) in one minute [18].

#### 2.4.4. Alkaline and Enzymatic Hydrolysis of the Solid Residues

The residues obtained from the HTP straw were further treated with 1% NaOH 20% at 50 °C or 30 °C for 48 h. Currently, most xylo-oligosaccharides are produced at the industrial level from enzymatic hydrolysis of alkaline-extracted xylan with xylanase.

Enzymatic hydrolysis of the solid residues was performed with 10% of residue (*w*/*v*) in 300 mL distilled water in Erlenmeyer flasks. The flasks were put in a a shaking incubator (Ecotron, Infors HT, Berlin, Germany) at 50 °C or 30 °C, for 48 h at 150 rpm. Commercial cellulase (Cellic^®^ CTec2, 100 FPU/mL) and Xylanase from *Trichoderma viride* (endo-1,4-β-Xylanase, 100–300 units/mg protein) were provided by Novozymes (Beijing, China) and Sigma-Aldrich (USA) and tested for saccharification experiments. The hydrolysates were monitored at specific time intervals and analyzed by HPLC using an UltiMate 3000 HPLC (Thermo Fisher DIONEX, Bavaria, Germany) with the column Eurokat H (KNAUER, Berlin, Germany). The dosages used for the saccharification experiments were as follows: Cellic^®^ CTec2: 4 FPU/g residue; 2 mL endo-b-1-4-xylanase from *T. viride* per 100 g residue. All the experiments were performed in duplicate, and the results were averaged.

#### 2.4.5. Filtration and Decolorization

The liquors were filtered manually using a 150 μm filter bag (Schwegmann Filtrations-Technik GmbH, Grafschaft, Germany) to remove the residues made of the lignocellulosic material. They were further centrifuged (4800 rpm 15 min—Sigma 4-16KS; Sigma Laborzentrifugen GmbH, Osterode am Harz, Germany) and decolorized using PUROLITE MN-502 (Purolite, Ratingen, Germany) or active charcoal. The flow was set to 6 bed volumes h^−1^. Decolorization was finished after rinsing the column with purified water until conductivity was below 1 mS cm^−1^.

#### 2.4.6. An- and Cation Exchange Chromatography

An- and cation exchange chromatography was performed in order to separate the sugars from the salt-ions. First, the weak anion exchange resin A 103 S (NH_3_ form, styrene-DVB) and second the strong cation exchange resin C 150 S (Na^+^ form, polystyrene- DVB) (Resindion S. R. L., Binasco, Italy) were applied. Before use, the resins were regenerated with the specific acid or alkaline solution, then carefully washed with distilled water. Column volumes were 1 L, and the loading was carried out from below at 6 bed volumes h^−1^. Adsorption experiments were conducted in flasks shaking at 150 rpm for 2 h at 50 °C, and the pH was adjusted with NaOH 20% and H_2_SO_4_. The pH and conductivity of the samples were measured. Phenol, kresol, catechol and guaiacol were chosen as representatives for phenols with inhibiting properties, obtained from Sigma-Aldrich (St. Louis, MO, USA).

The concentration of inorganic anions and cations were measured by ion chromatography using an ICS-1000 system (Thermo Scientific Dionex, Germering, Germany). For quantification of anions, 25 μL of sample volume was added on a IonPac AS9-HC column (4 × 250 mm) (Thermo Fisher DIONEX, Germany) and eluted isocratically with 1.2 ml/min^−1^ of 9 mM Na_2_CO_3_ at room temperature. For quantification of cations, 25 μL of sample volume was added on an IonPac CS 16 column (250 mm × 5) (Thermo Fisher DIONEX) and eluted isocratically with 1.0 mL min^−1^ of 30 mM CH_3_SO_3_H at 40 °C. Detection of cat- and anions was carried out by a conductivity cell. Each analysis was carried in duplicate, and peak areas were compared to analyses of known concentrations of salt-solutions consisting of the cat- and anions of interest. The mean concentration of the two analyses is presented.

The purified fraction was concentrated in a spray-dryer (Mobil minor; GEA, Düsseldorf, Germany) at a temperature between 120–135 °C, a pressure of 35 bar and a flow rate of approximately 10 mL/min.

### 2.5. Statistical Analysis

Statistical analysis was performed with IBM SPSS Statistics 19. All tests/experiments were conducted in triplicate, and the results were expressed as the means ± standard deviation (SD). Data normality was studied using the Shapiro-Wilk test [54], and the homogeneity of variances (homoelasticity) was studied using Levene test. A one-way ANOVA test was applied for normally distributed and homogeneous data. For *p* < 0.05 corresponding to statistical F, calculations with the Tukey post hoc test were used to study whether the treatments had significant differences. For non-homogeneous data, the Welch test was used, followed by the Kruskal–Wallis test, in which, if *p* < 0.05 was obtained, the calculations were continued with the Mann–Whitney test to verify which treatments had significant differences. At the parameters where the data were not homogeneous and we used Mann–Whitney, the Bonferroni correction was applied: 0.05/6 = 0.008 and 0.01/6 = 0.001; for 3 treatments, 0.05/3 = 0.016 and 0.01/3 = 0.003.

## 3. Results and Discussion

### 3.1. Chemical Characterization of Feedstock and Processed Solids

The tight structure of wheat straw cell wall is hard to hydrolyze without pretreatment processes that could overcome the recalcitrance of lignocellulose for the enzyme action. In this work, hydrothermal pretreatment (HTP), alkaline treatment and enzymatic treatment, or the combinations thereof, were used to improve the accessibility of the enzymes to the WS. Hydrothermal treatment was chosen due to the advantages highlighted in the literature, such as low cost, reduced chemical consumption due to the increase in acetic acid and water ionization that catalyze the process and facilitate depolymerization of the wheat straw structure, production of oligosaccharides and monosaccharides with decreased formation of degradation products, and the formation of residues that could further be fractioned [21].

Milling was applied to reduce the particle size and improve the hydrolysis by increasing the surface area [21]. The chemical composition of wheat straw is shown in Table 1. The holocellulose of wheat straw accounts for 68.67 ± 0.4% of the total weight and consists of 27.67 ± 0.1% hemicelluloses and 36% ± 0.3 celluloses. Lignin was quantified as acid detergent lignin 12.91 ± 0.8%, ash 5 ± 0.1% and extractives 1.2 ± 0.1%. During the HTP, the hemicelluloses decreased with the severity factor, from 24.7 ± 0.2% at 2.35 when treated at 140 °C for 15 min, to 11.3 ± 0.1% at a severity factor of 2.94 when HTP reached 180 °C. The dissolution of hemicellulose was caused by the ionization of water and formation of acetic acid [55]. Consequently, cellulose increased with the severity factor, from 45.12 ± 0.1% to 51 ± 0.5%. As for lignin, the content increased with the severity factor, with the highest content of 9.9% at 180 °C (Table 1). The pH profile after HTP shows the treatment severity and indicates the solubility of hemicelluloses in the liquid phase. The pH of the filtrate at the severity factor of 2.35 decreased from 5.2 to 4.5 at the severity of 2.94 (160 °C, 15 min) to 3.9 at the severity of 3.53 (180 °C, 15 min). Similar results were obtained by Chen et al. in 2018, who applied hydrothermal pretreatment combined with ethanol extraction to obtain oligosaccharides from wheat straws [10]. They observed that hemicellulose decreased with the HTP increasing temperature (from 27.06% *w*/*w* at 120 °C to 25.09% *w*/*w* at 140 °C and 3.09% *w*/*w* at 180 °C), while cellulose content increased with the increase of temperature from 120 °C to 180 °C.

### 3.2. Chemical Characterization of Hydrolysates after HTP, Alkaline and Enzymatic Treatments of the Solid Residues

Hydrothermal pretreatment was shown to degrade the hemicellulose present in the WS, improving the accessibility of cellulose to enzymes, with the formation of soluble sugars, xylose oligomers (xylo-oligosaccharides “XOS”), weak acids, furan derivatives and phenolic compounds. The results concerning the production of soluble sugars (glucose, xylose and arabinose) and xylo-oligosaccharides are presented in Table 2 and Table 3, respectively. In Supplementary File S1 the results obtained by other researchers in terms of wheat straw fractioning, sugars, xylo-oligosaccharides and inhibitor release are described.

#### 3.2.1. Sugar Release

**Figure 2:**
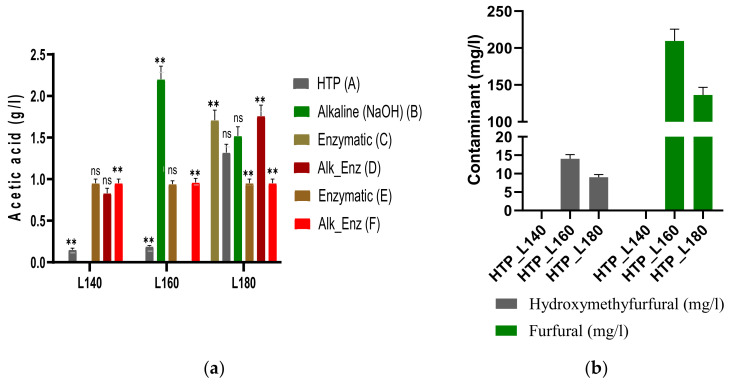
(**a**) Yield of acetic acid (g/L) detected in the hydrolysates after HTP, alkaline and enzymatic treatments of the solid residues; ns: not statistically significant; ** Correlation is significant at the 0.05 level (2-tailed); (**b**) Yield of furfural and HMF (mg/L) detected in the hydrolysates after HTP treatment.

The release of soluble sugars during HTP increased with the severity factor, from 0.17 ± 0.1 g/L at a severity factor of 2.35 to 0.89 ± 0.6 g/L at log R_0_ = 3.53. The depolymerization of hemicellulose occurred due to the hydrolytic action of the hydronium ions, which cleaved the acetyl groups to generate acetic acid [42]. The acetic acid content increased with the severity factor, from 0.15 ± 0.02 g/L at a severity factor of 2.35 (140 °C, 15 min) to 1.32 ± 0.01 g/L at a severity factor of 3.53 (180 °C, 15 min), as observed in Figure 2a. Huang et al. [18] observed the same trend after liquid hot water pretreatment of waste wheat straw for fermentable sugars and acetic acid, which increased with the rising temperature. Additionally, the authors obtained higher yields of sugars, with 15.8 g/L fermentable sugars at 180 °C for 40 min compared to our work, due to the different processing conditions. Chen et al. applied the same hydrothermal treatment as in our work but for longer residence time (0.5 h) and further applied an acid treatment with sulfuric acid to the hydrolysates to determine the oligosaccharide concentrations; they obtained higher yields of oligosaccharides than monosaccharides, which increased with the severity of the treatment from 2.49 g/kg at 120 °C to 61.69 g/kg at 180 °C [10]. Similarly, Ilanidis et al. reported comparable concentrations of monosaccharides to our work after HTP treatment of wheat straw at temperatures between 160 and 205 °C for 15 min (0.1 g/L xylose in the hydrolysate treated at 160 °C; 4.4 g/L xylose at the highest temperature of 205 °C) [56].

The solid residues after HTP were used to carry out the alkaline and enzymatic treatments. The alkaline treatment was shown to improve the removal of lignin and hemicellulose and swelling of the cellulose fibers [57]. Glucose release was highest at 160 °C (3.73 ± 0.28 g/L); however, the differences between the pretreatment temperatures were not statistically significant. Xylose and arabinose had the highest release at the highest severity factor (3.53) (3.80 ± 0.2 g/L and 0.59 ± 0.04 g/L, respectively) after the alkaline treatment method. Faryar et al. observed that xylose and arabinose were the main sugars extracted after alkaline treatment of wheat straw (0.047 g–0.056 g xylose/g WS) [58]. Akpinar et al. [59] applied an alkaline treatment (4% KOH, 1% *w*/*v* NaBH_4_) to extract xylan from different agricultural wastes (wheat straw, tobacco stalk, cotton stalk and sunflower stalk) and observed that WS had the highest amount of arabinose and a more arabinoxylan structure compared to the other biomasses.

Enzymatic hydrolysis was performed with two different enzymes for xylan and cellulose hydrolysis using Xylanase from *T. viride* and commercial cellulases (Cellic^®^ CTec2) in different concentrations. Alkaline enzymatic treatment was also applied to the residues to improve the accessibility of the enzymes, since lignin is soluble in highly alkaline media [57].

With regards to the enzymatic hydrolysis with xylanase, xylose release was highest in the alkaline hydrolysates of the residue treated at 180 °C, showing a quantity of 4.76 ± 0.35 g/L after 48 h of enzymatic hydrolysis at a pH of 4.9. When combining enzymatic with alkaline treatment, the xylose release almost doubled, showing a release of 9.47 ± 0.71 g/L in the hydrolysate of the residue treated at 180 °C. Glucose release was highest when the residues were treated with the enzymatic mix Cellic^®^CTec2, which contains aggressive cellulases, β-glucosidases and hemicellulases. Statistically significant differences were observed regarding conversion of cellulose to glucose after the different enzymatic treatments; the maximum glucose yield after 48 h of enzymatic hydrolysis with Cellic^®^CTec2 was found to be 10.72 ± 0.23 g/L at the autohydrolysis condition of 140 °C, which means about 19% of cellulose in the solid residue. Huang et al. used the same enzymatic cocktail to pretreat waste wheat straw (180 °C, 40 min) to recover soluble sugars (xylose, glucose) and obtained a recovery of maximum 33.4 g glucose and 6.1 g xylose after applying a washing treatment to remove the ash [18]. The authors also suggested that the ash in the residues conducted to reduced enzymatic hydrolysis efficiency; thus, the prewashing step was necessary. Han et al. reported that alkaline treatment (1%) improved the efficiency of enzymatic hydrolysis of wheat straw using a cellulase produced by *P. waksmanii* and obtained a maximal concentration of reducing sugar (343.95 mg/g substrate) [60]. Moreover, Silva-Fernandes et al. [61] applied an enzymatic hydrolysis to pretreated residues of WS by HTP (at 210 and 230 °C) and observed that the conditions applied for the enzymatic hydrolysis allowed a higher recovery of glucose (30.6 g/L glucose representing around 90% yield) from the residues pretreated at 230 °C compared to those at 210 °C (78.8%). They concluded that enzymatic hydrolysis allowed a high digestibility of cellulose fibers due to the physical degradation of lignocellulose, while diminishing the hemicellulose content in the residues and their inhibition towards cellulolytic enzymes [61]. When xylanase was used in the hydrolysis, the highest yield of glucose was detected in the hydrolysate at 180 °C, 6.67 ± 0.50 g/L, after enzymatic and alkaline treatment of the residues.

#### 3.2.2. Xylo-Oligosaccharide Production and Byproduct Release

In addition to monosaccharides, xylo-oligosaccharides with a low degree of polymerization (DP < 6) (xylotriose, X3, and xylotetraose, X4) were also detected in the hydrolysates after enzymatic and alkaline treatment of the residues. We observed that during the hydrothermal pretreatment, xylo-oligosaccharides were not detected, except a very low amount of xylobiose (0.01 g/L) in the hydrolysate at 180 °C, which might be caused by the autohydrolysis conditions, for instance, the long residence time (30–40 min) and pressure conditions (12 bar) in the reactor until it reached the desired temperatures. Guo et al. applied an acidolysis treatment with acetic acid to wheat straw and reported that the yield of released XOS was also dependent on the structural composition of the raw material, specifically the wax content and inert structure, which inhibit the production of XOS [42]. However, after the enzymatic and alkaline treatment of the residues, statistically significant differences were noticed. During the enzymatic treatment, xylanase was the most efficient in the hydrolysates obtained at 160 °C, with an amount of 1.23 ± 0.12 g/L XOS released, compared to the enzymatic mix, which showed an increasing trend of liberated XOS from 0.59 ± 0.03 g/L in the hydrolysates at 140 °C and at 160 °C to a maximum amount of 0.78 ± 0.03 g/L in the liquids at 180 °C. When combining enzymatic with alkaline treatment, which was shown to improve enzymatic digestibility, higher yields of XOS were obtained, showing statistically significant differences when compared to the enzymatic treatment. Xylotriose and xylotetraose were detected in the hydrolysates at 140 °C, with a content of 1.48 ± 0.2 g/L, and 1.33 ± 0.13 g/L in the filtrate at 180 °C, after 48 h of enzymatic hydrolysis with xylanase at pH 4.6. Faryar et al. reported concentrations of 1.94 ± 0.045–2.07 ± 0.068 mg XOS/mL (X2–X5) at pH 7 and 8, respectively, after enzymatic hydrolysis at 60 °C of wheat straw using an endoxylanase from *B. halodurans* [58]. Production of XOS from wheat straw was also studied by Akpinar et al., who reported 0.079 g XOS/g xylan after enzymatic hydrolysis with endoxylanase from *Aspergillus niger* [59].

Slightly lower yields were obtained when using the enzymatic mix Cellic CTec2, with an increasing trend of XOS being observed in the hydrolysates, from 1.11 ± 0.05 g/L in the hydrolysate at log R_0_ = 2.35 to a maximum of 1.26 ± 0.06 g/L in the hydrolysate at log R_0_ = 3.53. Huang et al. obtained higher yields of xylotryose and xylotetraose after enzymatic hydrolysis of pretreated wheat straw at 180 °C for 20 min (X3 + X4: xylotriose: 3.1 ± 0.7 g/L). The literature shows that XOS produced via alkaline enzymatic treatment have low aqueous solubility, since the acetyl and uronic groups are completely degraded [2,21].

During autohydrolysis, polysaccharides and monosaccharides could further decompose in degradation products like acetic acid, furfural and hydroxymethylfurfural (HMF). Figure 2a,b show the results of these degradation products. It was noticed that the yield of acetic acid increased with the severity of the hydrothermal treatment, from 0.15 ± 0.02 g/L to 1.32 ± 0.10 g/L in the hydrolysate at 180 °C. The highest content of acetic acid was detected after enzymatic treatment with xylanase and the combined alkali enzymatic with the same enzyme in the hydrolysates at 180 °C, with yields of 1.71 ± 0.12 g/L and 1.76 ± 0.13 g/L, showing statistically significant differences when compared to the other enzymatic treatment applied. Acetic acid originated from the hydrolysis of acetyl groups on the hemicellulose backbone, with the increase in the concentrations of byproducts leading to a decrease in the pH of the filtrate (from 5.2 to 3.9 after HTP). The results indicate a relationship between acid generation and hemicellulose solubilization, similar to other papers [43].

The other byproducts detected, furfural and HMF, resulted from the degradation of pentose (xylose and arabinose) and hexose (glucose); they were shown to hinder the enzymatic hydrolysis by inhibiting the activity of the enzyme and could inhibit the upgrade of both the liquid and solid fractions [10]. As observed in Figure 2a, the amounts of these metabolites released during the hydrothermal pretreatment increased with the severity factor from 2.35 to 3.53. For instance, furfural had a content of 209 ± 15.8 mg/L and HMF 14.81 ± 1.1 mg/L in the hydrolysate at log R_0_ = 2.94, while in the hydrolysate at log R_0_ = 3.53, furfural was detected in an amount of 136 ± 10 mg/L and HMF 9.54 ± 0.7 mg/L, due to the conversion of xylose and glucose. Chen et al. showed that furfural and HMF were detected only in the hydrolysate after pretreatment of wheat straw at 180 °C and 200 °C, with higher yields compared to our work (furfural: 8.4–17.6 g/kg and HMF: 0.3–4.5 g/kg) [10].

### 3.3. Material Balance

Material balances of hydrothermal pretreatment and subsequent alkaline, enzymatic and alkali enzymatic treatments are shown in Table 4. The material balance shows that the higher solid recovery of 88.9% was at the lowest severity of the hydrothermal pretreatment, and it decreased to 76.8% at the highest severity. The total amount of sugars released during HTP and alkaline treatment increased with the severity, from 2.35 (1.62 g/L) to 3.53 (8.76 g/L). It was noticed that the treatments applied showed high efficiency in releasing the sugars from the lignocellulosic materials, with the highest sugar recovery of 94% at the severity of 3.53 (180 °C, 15 min), obtained by considering the total amount of sugars released (glucose, xylose and arabinose) after combining all treatments applied (hydrothermal, alkaline, enzymatic hydrolysis and the alkali enzymatic). Discriminating between treatments, it was noticed that the hydrothermal pretreatment at the highest severity combined with enzymatic hydrolysis with xylanase had the highest sugar recovery of 64.86%, followed by the combined alkaline and enzymatic treatment with the enzymatic mix (Cellic^®^CTec2), which showed a recovery of 54.41%. In contrast, the lowest sugar recovery (18.79%) was shown at the lowest severity (140 °C), after enzymatic hydrolysis with xylanase.

### 3.4. Downstream Processing

In order to obtain a purified fraction of XOS for potential food applications, the hydrolysates obtained during hydrothermal pretreatment and enzymatic treatments must be refined. A variety of compounds like monosaccharides, acetic acid, furfural and HMF from pentose and hexose dehydration, soluble inorganic compounds or protein-derived products could appear in the hydrolysates [34]. Commercial xylo-oligosaccharides have a purity in the range of 75–95% [12]. The literature shows that purification and separation of XOS requires several processing steps, consisting of physicochemical treatments [62].

Adsorption was employed in combination with other treatments for the refining of XOS, intended to remove undesired compounds and separate oligo from monosaccharides. Chromatographic separation was carried out for XOS purification at an analytical level, yielding high purity fraction, while ion exchange was used for purification of XOS alone or in multi-step processing [12,34,62,63].

In this work, refining experiments were carried out in two steps: a first step of adsorption using the surface-active material activated charcoal or the polymer adsorber resin PUROLITE MN-502 to remove phenols and reduce the concentration of salt ions. The hydrolysate resulting after alkaline and enzymatic hydrolysis of the residues treated at the severity factor of 3.53 was used for the downstream processing experiments. As observed in Table 5, statistically significant differences were obtained when comparing removal of sulphate, phosphate and cations (K^+^, Mg^2+^, Ca_2_^+^, NH_4_^+-^N) by activated charcoal or the MN-502 resin, the latter showing higher efficiency. For instance, phosphate concentrations reduced from the initial 59 mg/L in the hydrolysate to 16 mg/L or 23 mg/L after 2 h of decolorization with the resin or the activated charcoal, respectively. Sulphate concentrations dropped almost to half, from 80 mg/L to 40 mg/L after treatment with the resin, showing statically significant differences when compared to the filtrate resulting after decolorization with charcoal (54 mg/L). Good results were obtained also in the case of HMF and furfural removal, which showed a decrease from 9.7 mg/L to 0.5 mg/L (MN 502) and <0.004 mg/L (charcoal) in the case of HMF. Furfural decreased from 139 mg/L to 2.3 mg/L after treatment with MN502 and <0.01 mg/L after charcoal treatment. In contrast, phenol concentrations did not decrease significantly. Monosaccharide concentrations were also reduced, with xylose being lowered from 9.4 mg/L to 1.3 mg/L after treatment with the resin and 0.09 mg/L after charcoal treatment. XOS registered a slight decrease, from 1.33 mg/L in the initial hydrolysate to 1.31 mg/L in the hydrolysate treated with the resin and to 1.26 mg/L in the hydrolysate treated with charcoal. Xu et al. used activate carbon treatment at different dosages (0.2%, 0.4%, 0.5%, 0.8%, 1% and 1.2%) to purify wood chip hydrolysates and observed that at higher dosage (1.2%), a loss of 20% xylosugars was noticed (1.5% xylose, 21% XOS loss), even if lignin and furfural were removed at a high percentage 70% [64]. It was reported that the adsorption behavior of the materials was affected by the molecular structure and weight [65].

An- and cation exchange chromatography was performed to further reduce the concentration of salts and minerals. It was previously shown that this method was successful in removing salt ions and other undesirable compounds and obtaining purified fractions of specific compounds such as lactic acid [66,67,68,69,70]. The anion exchange resin was more efficient in reducing the concentrations of sulphate, phosphates and nitrate, showing statistically significant differences (1.36 ± 0.01 mg/L PO_4_^3^—P vs. the initial content of 58.58 ± 0.50 mg/L PO_4_^3^—P; 24.3 ± 0.04 mg/L SO_4_^2−^ vs. the initial content of 80.82 ± 0.50 mg/L SO_4_^2−^). Dupoiron et al. showed that a weak anion exchange resin (Amberlyst A2) was successful in removing carbohydrate fractions, chlorides and other anions such as sulfates and phosphates from ferulic acid contained in wheat bran, due to the resin strong affinity for OH- [71].

Cation concentrations were lowered after the cation exchange, showing statistically significant differences; however, sodium concentrations increased significantly (507.22 ± 38 mg/L). Levels of HMF, furfural and phenols were also reduced during the downstream process (Table 6). Monosaccharide (xylose and arabinose) removal was the most efficient after the activated charcoal adsorption (92%). It was noticed that XOS concentrations registered a slight decrease during the down-streaming process; however, no statistically significant differences were noted.

Finally, it should be noted that the optimization of the downstream processing was not aim of the present work but is certainly needed to create feasible processes.

## 4. Conclusions

Our work showed that wheat straw could represent a low-cost alternative for production of fermentable sugars and xylo-oligosaccharides with low DP. Among the treatments applied, alkaline and enzymatic treatment with xylanase showed better results in terms of sugar release (glucose, xylose and arabinose) (18 g/L sugars), while XOS were generally released in lower amounts (highest concentration of 1.33 g/L). Refining experiments were carried out to obtain a purified fraction for potential food applications by using anion and cation exchange chromatography. The polymer adsorber resin MN-502 showed efficient removal of salts, cations, phenols and furan derivatives. However, further optimization treatments targeting a more efficient fractioning of the wheat straw are needed to obtain higher XOS yields in a purified form. Therefore, the integrated strategies in sugar-based biorefineries should target maximal sugar recoveries and fractioning processes that facilitate further conversion processes [72].

## Figures and Tables

**Table 1:** Chemical composition (% DM) of the raw material (WS) and pre-treated residues after the hydrothermal pretreatment.

	WS	R_140_	R_160_	R_180_
Severity factor		2.35	2.94	3.53
Hemicelluloses	27.67 ± 0.1	24.7 ± 0.2	25.3 ± 1	11.3 ± 0.1
Celluloses	36.0 ± 0.3	45.12 ± 0.1	49.6 ± 0.1	51.1 ± 0.5
Lignin (ADL ^a^)	12.91 ± 0.8	6.1 ± 0.4	9.67 ± 0.8	9.90 ± 1
Holocellulose	68.67 ± 0.4	69.828 ± 0.3	74.9 ± 1	62.4 ± 0.6
Ash	5.0 ± 0.1	1.2 ± 0.1	1 ± 0.2	0.8 ± 0.1
Extractives	1.2 ± 0.1	0.6 ± 0.1	0.8 ± 0.2	1.4 ± 0.1
Others	17.22 ± 0.1	11.18 ± 0.1	1.47 ± 0.1	2.3 ± 0.2
Yield	100	88.9 ± 0.8	87.84 ± 1.4	76.8 ± 0.7

^a^ ADL: acid detergent lignin; R140: residues after the HTP at 140 °C; R160: residues after the HTP at 160 °C; R180: residues after the HTP at 180 °C.

**Table 2:** Sugar yields in the hydrolysates after HTP, alkaline and enzymatic treatments of the solid residues.

	Treatment		Glucose (g/L)	Xylose (g/L)	Arabinose (g/L)	Total Sugars (g/L)
**L_140_**	HTP (A)		ND	ND	0.17 ± 0.1 NS (0.05)	0.17
	Alkaline (NaOH)(B)		0.49 ± 0.03 NS (0.393)	0.88 ± 06 NS (0.681)	0.08 ± 0.01 NS (0.05)	1.46
	Enzymatic	Xylanase (C)	0.88 ± 0.02 **	0.64 ± 0.48 **	0.28 ± 0.02 NS (0.05)	1.81
		CCTec2 (E)	10.72 ± 0.23 **	4.98 ± 0.26 NS (0.071)	0.49 ± 0.02 **	16.19
	Alkaline and enzymatic	Xylanase (D)	3.43 ± 0.02 **	4.4 ± 0.33 NS (0.075)	1.66 ± 0.11 **	9.49
		CCTec2 (F)	9.96 ± 0.52 **	4.97 ± 0.26 **	0.49 ± 0.02 *	15.42
**L_160_**	HTP (A)		ND	0.22 ± 0.08 **	0.42 ± 0.28NS (0.513)	0.64
	Alkaline (NaOH) (B)		3.73 ± 0.28 NS (0.07)	3.38 ± 0.25 NS (0.89)	0.48 ± 0.03NS (0.513)	7.59
	Enzymatic	Xylanase (C)	4.40 ± 0.31 **	3.43 ± 0.25 **	1.03 ± 0.59 *	8.86
		CCTec2 (E)	2.70 ± 0.14 **	2.08 ± 0.11 **	0.25 ± 0.01 *	5.03
	Alkaline and enzymatic	Xylanase (D)	5.39 ± 0.40 **	2.94 ± 0.22 **	1.02 ± 0.07 *	9.35
		CCTec2 (F)	1.99 ± 0.10 **	1.66 ± 0.08 **	0.21 ± 0.01 NS (0.513)	3.86
**L_180_**	HTP (A)		ND	0.10 ± 0.00 **	0.79 ± 0.06 NS (0.899)	0.89
	Alkaline (NaOH) (B)		3.48 ± 0.26 NS (0.317)	3.80 ± 0.20 NS (0.061)	0.59 ± 0.04 **	7.87
	Enzymatic	Xylanase (C)	2.79 ± 0.21 **	4.76 ± 0.35 NS (0.899)	3.55 ± 0.26 **	11.1
		CCTec2 (E)	9.37 ± 0.49 **	4.50 ± 0.23 **	0.43 ± 0.02 **	14.3
	Alkaline and enzymatic	Xylanase (D)	6.67 ± 0.50 **	9.47 ± 0.71 **	1.54 ± 0.64 **	17.68
		CCTec2 (F)	9.38 ± 0.49 **	4.50 ± 0.23 **	0.42 ± 0.02 NS (0.611)	14.3

ND: not determined; NS: not statistically significant; * Correlation is significant at the 0.05 level (2-tailed); ** Correlation is significant at the 0.01 level (2-tailed); L_140_: liquid hydrolysates of the residues treated at 140 °C; L_160_: liquid hydrolysates of the residues treated at 160 °C; L_180_: liquid hydrolysates of the residues treated at 180 °C.

**Table 3:** Xylo-oligosaccharides yields in the hydrolysates after HTP, alkaline and enzymatic treatments of the solid residues.

			Xylotriose (g/L)	Xylotetraose (g/L)	Total XOS (g/L)
L140	HTP (A)		ND	ND	ND
	Alkaline (NaOH) (B)		ND	ND	ND
	Enzymatic	Xylanase (C)	ND	ND	ND
		CCTec2 (E)	0.57 ± 0.03 **	0.02 ± 0.001 **	0.59 ± 0.03 **
	Alkaline and enzymatic	Xylanase (D)	1.32 ± 0.1 **	0.13 ± 0.01 **	1.48 ± 0.2 **
		CCTec2 (F)	1.07 ± 0.05 **	0.04 ± 0.002 NS (0.016)	1.11 ± 0.05 **
L160	HTP (A)		ND	ND	ND
	Alkaline (NaOH) (B)		ND	ND	ND
	Enzymatic	Xylanase (C)	1.12 ± 0.08 **	0.08 ± 0.01	1.23 ± 0.12 **
		CCTec2 (E)	0.52 ± 0.02 **	0.07 ± 0.00	0.59 ± 0.03 **
	Alkaline and enzymatic	Xylanase (D)	ND	ND	ND
		CCTec2 (F)	1.14 ± 0.06 NS (0.899)	0.08 ± 0.00	1.23 ± 0.05 NS (0.899)
L180	HTP (A)		ND	ND	0.01 ± 0.00 **
	Alkaline (NaOH) (B)		ND	ND	ND
	Enzymatic	Xylanase (C)	ND	ND	ND
		CCTec2 (E)	0.76 ± 0.04 **	0.01 ± 0.00 **	0.78 ± 0.03 **
	Alkaline and enzymatic	Xylanase (D)	1.25 ± 0.09 NS (0.754)	0.04 ± 0.00 NS (0.797)	1.33 ± 0.13 NS (0.721)
		CCTec2 (F)	1.21 ± 0.06 **	0.04 ± 0.00 **	1.26 ± 0.06 **

NS: not statistically significant; ND: not determined; ** Correlation is significant at the 0.01 level (2-tailed).

**Table 4:** Material balances from hydrothermal pretreatment followed by alkali, enzymatic and combined alkali enzymatic treatments.

Temperature (°C)	Severity Factor	Solid Recovery (%)	Sugars in Filtrates (HTP and Alkaline) (g)				Enzyme Hydrolysates (Combined Enzymatic and Alkali Enzymatic Treatments) (g)				Sugar Recovery	
			G ^a^	X ^b^	A ^c^	T ^d^	G ^a^	X ^b^	A ^c^	T ^d^	(g) ^e^	(%) ^f^
140	2.35	88.9	0.49	0.88	0.25	1.62	24.98	14.99	2.92	42.89	44.5	64.8
160	2.94	87.84	3.73	3.6	0.9	8.23	14.48	10.11	2.51	27.1	35.3	51.4
180	3.53	76.8	3.48	3.90	1.38	8.76	28.21	23.23	4.44	55.88	64.6	94

^a^ G: released glucose; ^b^ X: released xylose; ^c^ A: released arabinose; ^d^ T: total sugars; ^e^ sum of sugars in the hydrolysates + alkaline and combined enzymatic and alkali hydrolysates; ^f^ Percentage of sugar recovery, calculated by (sugar recovery (g)/carbohydrate in the raw material (g)).

**Table 5:** Overview of the down-stream processing experiments of 1 L of hydrolysate after alkaline and enzymatic treatment of the residues treated at the highest severity factor (3.53).

Parameters		Resins			
Xylose (g/L)	Hydrolysate D severity factor (log R_0_ = 3.53)	MN-502	AC	A103S	C150S
	9.47 ± 0.01NS (0.992)	1.31 ± 0.01NS (1.00)	0.09 ± 0.01 *	1.29 ± 0.00 **	1.28 ± 0.00 **
Arabinose (g/L)	1.54 ± 0.64 **	0.78 ± 0.05NS (1.00)	0.79 ± 0.05 **	0.18 ± 0.00NS (0.05)	0.05 ± 0.00 NS (0.05)
Monosaccharides removal (%)		81.01	92.0	86.64	87.92
XOS (g/L)	1.33 ± 0.13 NS (0.721)	1.31 ± 0.06 NS	1.26 ± 0.03 NS	1.19 ± 0.04 NS	1.1 ± 0.01 NS
Acetic acid (g/L)	1.76 ± 0.05 **	0.27 ± 0.01 NS (1.00)	0.2 ± 0.01 NS (1.00)	0.18 ± 0.01 NS (1.00)	0.35 ± 0.01 NS (0.05)
PO_4_^3^--P (mg/L)	58.58 ± 0.50 **	16.34 ± 1.23 **	23.73 ± 1.79 **	1.36 ± 0.01 **	11.09 ± 0.83 **
SO_4_^2^- (mg/L)	80.82 ± 0.50 **	40.04 ± 3.02 **	54.73 ± 4.14 **	24.3 ± 0.04 **	31.76 ± 2.40 **
NO_3_-N (mg/L)	3.82 ± 0.10 **	1.39 ± 0.10 NS (0.872)	1.49 ± 0.11 *	0.19 ± 0.1 *	1.09 ± 0.08 *
Na^+^ (mg/L)	31.40 ± 0.60 NS (0.827)	30.41 ± 2.29 NS (0.05)	41.50 ± 3.13 **	30.54 ± 1 *	507.22 ± 38.34 **
K^+^ (mg/L)	562.69 ± 1.50 **	5.95 ± 0.45 **	190.83 ± 14.42 **	426 ± 1.1 *	5.33 ± 0.39 **
Mg^2+^ (mg/L)	61.74 ± 0.50 **	0.44 ± 0.03 **	21.15 ± 1.59 **	55.07 ± 0.5 *	4.26 ± 0.32 **
Ca_2_^+^ (mg/L)	95.51 ± 0.50 **	3.95 ± 0.29 **	28.84 ± 2.18 **	66.44 ± 0.1 *	10.03 ± 0.75 **
NH_4_^+-^N (mg/L)	42.47 ± 1.01 **	0.23 ± 0.01 **	14.88 ± 1.12 **	21.29 ± 0.27 *	4.20 ± 0.31 **

At the parameters where the data were not homogeneous and we used Mann–Whitney, the Bonferroni correction was applied: 0.05/5 = 0.01 and 0.01/5 = 0.002; NS: not statistically significant; * Correlation is significant at the 0.05 level (2-tailed); ** Correlation is significant at the 0.01 level (2-tailed).

**Table 6:** Removal of furfural, HMF and phenols after the down-streaming process.

Parameters		Resins			
Parameter	Hydrolysate D Severity Factor 3.53	MN502	AC	A103S	C150S
HMF (mg/L)	9.71 ± 0.02	0.50 ± 0.01	<0.004	0.71 ± 0.01	0.65 ± 0.01
Furfural (mg/L)	139 ± 0.3	2.31 ± 0.1	<0.0124	<0.0124	5.37 ± 0.1
Phenol (mg/L)	16.66 ± 0.02	<0.079	<0.079	<0.079	16.20 ± 0.01
Kresol (mg/L)	<0.058	<0.058	<0.058	<0.058	<0.058
Catechol (mg/L)	<0.027	<0.027	<0.027	<0.027	<0.027
Guajacol (mg/L)	3.81 ± 0.01	<0.008	<0.008	<0.008	<0.008

## Data Availability

The data presented in this study are available within the article. Other data that support the findings of this study are available upon request from the corresponding authors.

## Supplementary Materials

The following supporting information can be downloaded at: https://www.mdpi.com/article/10.3390/polym14071336/s1, Supplementary File S1. State of the art regarding wheat straw fractioning after various treatments in terms of monosaccharides, xylo-oligosaccharides and inhibitors yields.

## Abbreviations

XOS	Xylo-oligosaccharides
X3	Xylotriose
X4	Xylotetraose
SCFA	Short-chain fatty acids
WS	Wheat straw
HTP	Hydrothermal pretreatment
HMF	Hydroxymethylfurfural
DP	Degree of polymerization
DNS	Dinitrosalicylic method
CCTec2	Cellic CTec 2 enzyme
ADL	acid detergent lignin
NS	Not statistically significant
ND	Not determined
AC	Active charcoal
