# Supplementary material for: Chemical and Enzymatic Synthesis of Biobased Xylo-Oligosaccharides and Fermentable Sugars from Wheat Straw for Food Applications
Source: Polymers (Basel). 2022 Mar 25;14(7):1336. doi: 10.3390/polym14071336 (PMC9003230; doi:10.3390/polym14071336)
Supplement: Supplementary file 1 [file polymers-14-01336-s001.zip › polymers-1599954-supplementary.pdf]

Supplementary File S1. State of the art regarding wheat straw fractioning after various treatments in terms of monosaccharides, xylo-oligosaccharides and inhibitors yields

Reference	Treatment Conditions	Samples[3]	Monosaccharides			Xylo-oligosaccharides (XOS)					Inhibitors		
			**Glucose (g/kg)**	**Xylose (g/kg)**	**Total Sugars (g/kg)**	**Xylobiose (X2) (g/kg)**	**Xylotriose (g/kg)**	**Xylotetraose (g/kg)**	**Other XOS (DP 4–6 or DP > 6) (g/kg)**	**Total XOS (g/kg)**	**Acetic Acid (g/kg)**	**Furfural (g/kg)**	**HMF (g/kg)**
Chen et al., 2018 [10]	HTP[1] (120, 140, 160, 180 and 200 °C, 30 min); Alkaline ethanol treatment (NaOH), enzymatic hydrolysis (CellicCTec2), 50 °C, 72 h	L_{140}	NA[8]	0.06		0.11	0.07	0.05	4.59	4.82	10.9	ND	ND
		L_{160}	NA	0.21		0.58	0.59	0.30	30.15	31.62	13.99	ND	ND
		L_{180}	NA	6.04		5.37	4.25	2.27	49.8	61.69			
Huang et al., 2017 [18]	LHWP[2] (140–220 °C, 20–80 min)	$L_{140\ (40\ min)}$	ND[9]	10± 0.0				5 ± 0.1			0.6 ± 0.2	0.0 ± 0.0	
		$L_{160\ (40\ min)}$	1 ± 0.0	2 ± 0.0				20 ± 0.2			0.8 ± 0.1	0.0 ± 0.0	
		$L_{180\ (20\ min)}$	3 ± 0.2	2 ± 0.0				42 ± 0.5			0.8 ± 0.0	0.1 ± 0.0	
		$L_{180\ (40\ min)}$	3 ± 0.1	6 ± 0.2				49 ± 0.2			1.0 ± 0.1	0.1 ± 0.0	
		$L_{180\ (60\ min)}$	2 ± 0.1	4 ± 0.1				48 ± 0.4			1.0 ± 0.3	0.2 ± 0.1	
		$L_{180\ (80\ min)}$	2 ± 0.0	5 ± 0.0				36 ± 0.1			1.1 ± 0.3	0.2 ± 0.0	
	Enzymatic hydrolysis ((Cellic CTec2 (25 FPU/ g-cellulose), 50 °C, 150 rpm 48 h))	$L_{140\ (40\ min)}$	65 ± 0.1	32 ± 0.2									
		$L_{160\ (40\ min)}$	70 ± 0.0	41 ± 0.9									
		$L_{180\ (20\ min}$	90 ± 0.4	42 ± 0.2		1.5 ± 0.1	1.4 ± 0.1	1.7 ± 0.6	0.17 ± 0.0	9.2 ± 0.2			
		$L_{180\ (40\ min)}$	117 ± 0.7	41 ± 0.5									
		$L_{180\ (20\ min)}$	90 ± 0.4	42 ± 0.2									
		$L_{180\ (60\ min)}$	116 ± 0.7	31 ± 0.3									
		$L_{180\ (80\ min)}$	122 ± 0.3	26 ± 0.5									
Ertas et al., 2014 [15]	HTP (160–200 °C for 10–20 min)+acid hydrolysis ((4% (w/w) H2SO4 for 1 h at 121 °C)	$L_{160\ (10\ min)}$	1.3	1.5[4]	2.8								
		$L_{160\ (20\ min)}$	1.8	2.2	4.0								
		$L_{180\ (10\ min)}$	2.9	6.8	9.7								
		$L_{180\ (20\ min)}$	2.3	8.5	10.8								
		$L_{190\ (10\ min)}$	2.9	9.3	12.2								
		$L_{190\ (20\ min)}$	2.7	7.4	10.1								
Antov and Dordevic, 2017 [17]	US[5] +enzymatic hydrolysis (endo-xylanase, 0.15 U/g or 0.30 U/g, 48h	L1[6]	30.9	7.9				85.1					
		L2[7]	37.4	14.2				83.8					
Ilanidis et al., 2021 [56]	HTP (160, 175, 190, and 205 °C, 15 min)	L_{160}	0.1 ± 0.1	0.1± 0.1				NA			15 ± 0.1	<0.1 ± 0.1	<0.1 ± 0.1
		L_{175}	0.1 ± 0.1	0.1 ± 0.1				NA			22.5 ± 0.1	<0.1 ± 0.1	<0.1 ± 0.1
		L_{190}	0.1 ± 0.1	1.6 ± 0.1				NA			36.1 ± 0.1	<0.1 ± 0.1	<0.1 ± 0.1
		L_{205}	0.7 ± 0.1	4.4 ± 0.1									
	aqueous sulfuric acid (160, 190°C, 15 min)	L_{160}	0.2 ± 0.1	0.1 ± 0.1				NA			11.7 ± 0.1	<0.1 ± 0.1	<0.1 ± 0.1
		L_{190}	0.2 ± 0.1	2.5 ± 0.1				NA			33.8 ± 0.1	<0.1 ± 0.1	<0.1 ± 0.1
Akpinar et al., 2009 [59]	Alkaline treatment (xylan extraction) Enzymatic hydrolysis (4U/mL of xylanase from *A. niger*, pH 5.5 40 °C, 24h or 4U/mL of xylanase from *T. longibrachiatum* , pH 4.6 at 50 °C, 24h)[10]		3.15 ± 0.07	79.9 ± 0.2		0.786 ± 0.079	0.719 ± 0.072	0.0767 ± 0.008	0.551	0.719			
Carvalheiro et al., 2009 [43]	Autohydrolysis (150 °C–240 °C, non-isothermal conditions)	L_{150}	0.63	1.66							0.64	ND	0.01
		L_{170}	0.97	1.79							0.80	ND	0.01
		L_{190}	0.73	1.44							1.31	0.01	0.01
		L_{200}	1.63	0.84							1.70	0.03	0.01
Nabarlatz et al., 2007 [20]	Authohydrolysis (179 °C, 23 min)[11]		0.8	0.9						41.2	0.6	0.0	0.2
